# Salud pública y cáncer cervicouterino en América Latina: impacto del gasto y legislación en la prevención en tres países

**DOI:** 10.1016/j.aprim.2025.103427

**Published:** 2026-01-14

**Authors:** Camilo Andrés Estupiñan Ruiz, Rubén Darío Morales Doncel, Jeisson Andrés Hincapié-Carvajal

**Affiliations:** a Fisioterapia en cuidados críticos, Salud Pública, Universidad Nacional de Colombia, Investigaciones y Proyección Social, Facultad de Enfermería, Universidad El Bosque, Bogotá, Colombia; b Especialidad en Bioestadística, Bogotá, Colombia; c Universidad El Bosque, Bogotá, Colombia

**Keywords:** Salud pública, Cáncer, Cáncer cervicouterino, Public health, Cancer, Cervical cancer

## Abstract

**Objetivo:**

Evaluar la asociación entre gasto público en salud, marco legal e indicadores de incidencia, mortalidad y esperanza de vida femenina por cáncer de cuello uterino en Chile, Colombia y Nicaragua entre 2012 y 2022.

**Diseño:**

estudio ecológico descriptivo y correlacional con datos agregados nacionales.

**Emplazamiento:**

Tres países latinoamericanos: Chile, Colombia y Nicaragua.

**Participantes:**

Población femenina general registrada en bases de la Organización Panamericana de la Salud (OPS).

**Intervenciones:**

Revisión de marcos normativos y políticas públicas, incluyendo programas de cribado, vacunación y mecanismos de financiación sanitaria.

**Mediciones principales:**

Gasto público en salud (% del PIB), esperanza de vida femenina al nacer, incidencia y mortalidad por cáncer de cuello uterino (por 100.000 mujeres). Se calcularon medias decenales (2012-2022) y correlaciones de Pearson.

**Resultados:**

En 2022, Chile presentó la mayor esperanza de vida (82,0 años) y la menor mortalidad (11,3/100.000) con gasto del 8,9% del PIB. Colombia mostró 79,6 años, mortalidad de 12,7/100.000 y gasto de 6,4%. Nicaragua tuvo la menor inversión (5,8%), esperanza de vida más baja (74,8 años) y mayores tasas de incidencia (20,6) y mortalidad (11,5). Se hallaron correlaciones negativas muy fuertes entre esperanza de vida e incidencia (r = −0,97) y mortalidad (r = −0,81), y relación débil con el gasto (r ≈ −0,13).

**Conclusiones:**

Más que el monto invertido, la efectividad depende del marco legal y de la orientación estratégica. Se recomienda fortalecer la legislación, priorizar la atención primaria y ampliar la cobertura de cribado y vacunación para eliminar esta enfermedad.

## Abstract (en)

**Objective:**

To assess the association between public health expenditure, legal frameworks, and indicators of cervical cancer incidence, mortality, and female life expectancy in Chile, Colombia, and Nicaragua from 2012 to 2022.

**Design:**

Descriptive and correlational ecological study based on nationally aggregated data.

**Setting:**

Three Latin American countries with information from the Pan American Health Organization (PAHO).

**Participants:**

General female population.

**Interventions:**

Review of national health legislation and policies, including screening, vaccination, and financing mechanisms.

**Main measurements:**

Public health expenditure (% GDP), female life expectancy at birth, and cervical cancer incidence and mortality rates per 100,000 women. Ten-year averages and Pearson correlation coefficients were calculated.

**Results:**

In 2022, Chile showed the highest life expectancy (82.0 years) and the lowest mortality (11.3/100,000) with spending of 8.9% of GDP. Colombia reached 79.6 years of life expectancy, mortality of 12.7/100,000, and spending of 6.4%, while Nicaragua had the lowest investment (5.8%), lowest life expectancy (74.8 years), and highest incidence (20.6) and mortality (11.5). Strong negative correlations were found between life expectancy and incidence (r = −0.97) and mortality (r = −0.81), but weak correlation with public spending (r ≈ −0.13).

**Conclusions:**

The effectiveness of cervical cancer prevention programs depends less on investment volume than on legal strength and strategic direction. Strengthening legislation, prioritizing primary care, and expanding screening and vaccination are key to eliminating this preventable disease in Latin America.

## Introducción

América Latina y el Caribe han logrado avances notables en la ampliación de la cobertura y el acceso a servicios básicos de salud, así como en el fortalecimiento de la atención primaria. Sin embargo, persisten retos estructurales, como la fragmentación del financiamiento, la duplicidad de esquemas de aseguramiento y la desigual distribución de recursos humanos y tecnológicos entre zonas urbanas y rurales. Estas brechas impactan la efectividad de las intervenciones preventivas frente a enfermedades evitables, como el cáncer de cuello uterino, generando desigualdades en la detección y el tratamiento^1^, ^2^.

En 2020, la Organización Mundial de la Salud (OMS) lanzó la Estrategia Global para la Eliminación del Cáncer Cervical, que propone alcanzar el 90% de cobertura de vacunación contra el VPH en niñas, el 70% de cribado con pruebas de alta precisión en mujeres y el 90% de tratamiento adecuado en casos detectados (Muñoz y Bravo^2^, 2022). La Organización Panamericana de la Salud (OPS) adoptó este marco en su Plan de Acción 2020-2030, destacando que para cumplir estas metas se requieren sistemas de salud resilientes, integrados y equitativos^3^, ^4^. A pesar de este impulso global, los países de la región presentan notoria heterogeneidad en la asignación del gasto público en salud —entre el 5% y el 9% del PIB— y en la solidez de sus marcos normativos para la prevención y el control del cáncer cervicouterino^5^. Los estudios existentes suelen abordar de forma aislada aspectos como la cobertura del cribado o el impacto de políticas nacionales específicas, sin integrar simultáneamente variables financieras, regulatorias y de resultados poblacionales comparados^2^, ^3^, ^5^, ^6^, ^7^, ^8^.

Los sistemas de salud latinoamericanos difieren ampliamente en estructura, cobertura, financiamiento y acceso. En Chile, el sistema mixto combina el sector público (FONASA) y el privado (ISAPRE) bajo regulación estatal. Este país ha desarrollado políticas sólidas de atención a enfermedades crónicas, y desde 2005 el cáncer cervicouterino está cubierto por el régimen AUGE, garantizando diagnóstico y tratamiento oportuno^9^.

Colombia también posee un sistema mixto, basado en aseguramiento universal mediante regímenes contributivo y subsidiado. Su Programa Nacional para el Control de Cáncer de Mama y Cuello Uterino y el Fondo Colombiano de Enfermedades de Alto Costo^10^, ^11^ han fortalecido la detección temprana y la monitorización de indicadores, aunque persisten desigualdades territoriales y de acceso^2^, ^7^, ^12^, ^13^, ^14^, ^15^, ^16^.

Nicaragua, por su parte, ha transitado hacia un modelo familiar y comunitario, con enfoque intercultural y gratuito. A diferencia de los esquemas de aseguramiento, el Estado asume directamente la provisión de servicios, lo que ha ampliado la cobertura, aunque persisten limitaciones tecnológicas y diagnósticas. En cáncer cervicouterino, destacan campañas de cribado junto con la Fundación Movicáncer^5^ y la creación de rutas integrales de atención (Ministerio de Salud de Nicaragua, 2020). Pese a ello, las tasas de incidencia y mortalidad siguen siendo elevadas^3^, ^4^, ^5^, ^17^, ^18^.

La comparación entre los tres países evidencia cómo la estructura del sistema de salud condiciona la efectividad de las políticas de prevención. Chile y Colombia han integrado el cribado dentro de paquetes de beneficios, mientras que Nicaragua enfrenta desafíos en calidad y equidad. En conjunto, los tres casos subrayan la necesidad de fortalecer la detección temprana y los marcos regulatorios como ejes centrales en la lucha contra una enfermedad prevenible que afecta desproporcionadamente a mujeres en contextos vulnerables^5^, ^6^, ^8^, ^18^, ^19^.

El objetivo de este estudio fue evaluar la asociación entre gasto público en salud (como porcentaje del PIB), marco normativo e indicadores de incidencia, mortalidad y esperanza de vida femenina por cáncer de cuello uterino en Chile, Colombia y Nicaragua durante 2012 a 2022. Este análisis busca aportar evidencia útil para orientar políticas efectivas y equitativas que aceleren la eliminación del cáncer cervicouterino en la región^3^, ^18^, ^19^.

## Materiales y métodos

### Diseño

Estudio ecológico descriptivo y correlacional basado en datos agregados a nivel nacional, con análisis comparativo entre tres países de América Latina (Chile, Colombia y Nicaragua) durante el periodo 2012-2022.

### Fuentes de datos

*Indicadores de salud:* portal de datos abiertos de la Organización Panamericana de la Salud (OPS), conjunto «Indicadores Básicos de Salud para América Latina y el Caribe» (2012-2022)^19^.

*Documentación normativa:* textos de leyes, decretos y planes nacionales de prevención de cáncer cervical disponibles en sitios oficiales de los países estudiados.

*Población y unidades de análisis:* mujeres de todas las edades para los indicadores de esperanza de vida, incidencia y mortalidad por cáncer de cuello uterino. Se usaron los datos agregados anuales por país.

Las variables e indicadores seleccionados aparecen en la tabla 1.

**Tabla 1:** Variables e indicadores seleccionados

Categoría	Indicador	Fuente OPS
Demográfica	Esperanza de vida al nacer femenina (años)	OPS, Indicadores Básicos (2021)
Financiera	Gasto público en salud (% del PIB)	OPS, Indicadores Básicos (2023)
Epidemiológica	Tasa de incidencia de cáncer de cuello uterino (por 100.000 mujeres)	OPS, Indicadores Básicos (2023)
	Tasa de mortalidad por cáncer de cuello uterino (por 100.000 mujeres)	OPS, Indicadores Básicos (2023)

### Procesamiento y limpieza de datos

*Descarga y estandarización:* importación de archivos CSV desde el portal OPS; unificación de nombres de variables y formatos de fecha.

*Manejo de datos faltantes:* se empleó estrategia de «pareamiento completo» *(complete-case analysis)* para los pares de indicadores en cada año y país, descartando únicamente los registros con al menos una variable ausente.

*Cálculo de medias decenales:* para visualizar tendencias generales, se calcularon las medias de cada indicador en el conjunto 2012-2022.

### Análisis estadístico

Realizado en RStudio (versión 4.x) con los siguientes paquetes: tidyverse: manipulación y transformación de datos; ggplot2: generación de gráficos de tendencia y dispersión; stats (base R): cálculo de coeficiente de correlación de Pearson.

### Procesamiento de la información

Para el análisis descriptivo se usó el cálculo de medias, medianas y rangos para cada indicador por país y año. Para los gráficos de tendencia temporal, las series de tiempo de cada variable (2012-2022) para identificar patrones y posibles cambios estructurales.

*Correlaciones bivariadas:* coeficiente de Pearson (r) para evaluar la fuerza y la dirección de la relación lineal entre: esperanza de vida vs. incidencia de cáncer cervical; esperanza de vida vs. mortalidad por cáncer cervical, y gasto público vs. incidencia y mortalidad. La interpretación de r fue según criterios convencionales: 0,00-0,19: muy débil; 0,20-0,39: débil; 0,40-0,59: moderada; 0,60-0,79: fuerte, y 0,80-1,00: muy fuerte.

### Revisión de marcos normativos

Se recopiló y resumió la legislación, los planes y los protocolos nacionales relacionados con prevención, cribado y tratamiento de cáncer de cuello uterino de cada país, para evaluarlos cualitativamente en función de: año de promulgación, alcance poblacional, garantías de cobertura, e instrumentos de seguimiento y evaluación.

## Resultados

**Figura 1:**
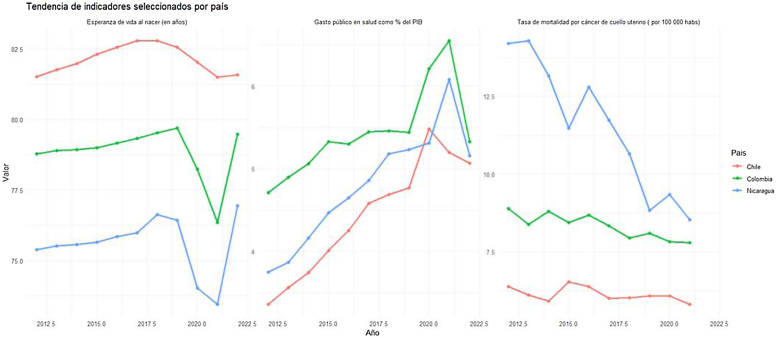
Tendencias temporales de indicadores clave de salud (2012-2022) en Chile, Colombia y Nicaragua.

La figura 1 muestra la tendencia temporal (2012-2022) de los indicadores seleccionados por país. Se observan diferencias marcadas: Chile mantuvo una esperanza de vida femenina alta (alrededor de 82 años), acompañada de un gasto público creciente. Colombia presentó un ascenso progresivo hasta 2019, seguido de una caída entre 2020 y 2021 atribuible a la pandemia de COVID-19. Nicaragua, en contraste, exhibió los valores más bajos de esperanza de vida y gasto, aunque con incremento sostenido en los últimos años.

**Figura 2:**
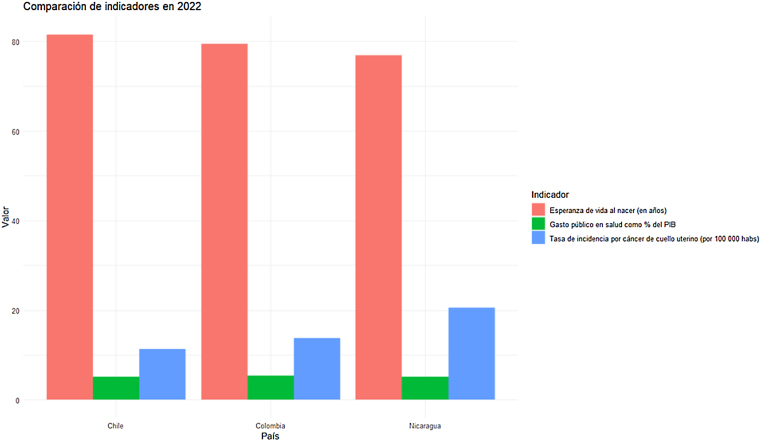
Comparación de indicadores de salud seleccionados en 2022 entre Chile, Colombia y Nicaragua.

La figura 2 corresponde a una comparación puntual en 2022, que permite visualizar el contraste entre los países en cada indicador. Nicaragua presentó la mayor incidencia de cáncer de cuello uterino (20,6 por 100.000), seguida de Colombia^7^, ^13^ y Chile^3^, ^11^. También registró la menor esperanza de vida y un gasto público intermedio.

La tabla 2 resume los principales estadísticos descriptivos (media, mediana, mínimos y máximos) por país e indicador. De forma general, se confirma que Chile lidera en longevidad, Colombia en gasto público, y Nicaragua enfrenta los mayores retos en cáncer cervicouterino, tanto en incidencia como en mortalidad.

**Tabla 2:** Resumen estadístico descriptivo de esperanza de vida, gasto público en salud e indicadores de cáncer cervicouterino

País	Indicador	Media	Mediana	Mínimo	Máximo	n
Chile	Esperanza de vida al nacer (en años)	82,1	82,03	84,5	82,9	5
Chile	Gasto público en salud como % del PIB	5,042	5,07	4,7	5,5	5
Chile	Tasa de mortalidad por cáncer de cuello uterino (por 100.000 hab.)	6,00	6,05	5,8	6,1	4
Colombia	Esperanza de vida al nacer (en años)	78,6	79,5	76,3	79,7	5
Colombia	Gasto público en salud como % del PIB	5,8	5,5	5,3	6,5	5
Colombia	Tasa de mortalidad por cáncer de cuello uterino (por 100.000 hab.)	7,9	7,9	7,8	8,1	4
Nicaragua	Esperanza de vida al nacer (en años)	75,5	76,4	73,4	76,9	5
Nicaragua	Gasto público en salud como % del PIB	5,4	5,2	5,2	6,1	5
Nicaragua	Tasa de mortalidad por cáncer de cuello uterino (por 100.000 hab.)	9,3	9,1	8,5	10,6	4

La tabla 3 presenta la matriz de correlaciones entre indicadores. Entre los principales hallazgos destacan: La relación entre gasto público y mortalidad por cáncer fue negativa pero débil (−0,13). La correlación entre gasto público e incidencia (solo 2022) fue prácticamente nula (0,075). Se observó una fuerte correlación negativa entre esperanza de vida e incidencia (−0,97), lo que sugiere que una mayor carga de cáncer prevenible se asocia con menor longevidad femenina. La correlación entre mortalidad y esperanza de vida fue fuertemente negativa (−0,81), respaldando el impacto directo de esta patología en los años de vida de las mujeres. Un hallazgo adicional fue la correlación negativa moderada entre gasto público y esperanza de vida (−0,53), lo que plantea interrogantes sobre la eficiencia y la orientación del gasto más allá de su volumen.

**Tabla 3:** Correlaciones entre gasto público, esperanza de vida e indicadores de cáncer de cuello uterino

Indicadores	Esperanza de vida	Gasto público	Mortalidad cuello uterino
Esperanza de vida en mujeres	1,00	–0,53	–0,81
Gasto público en salud (% PIB)	–0,53	1,00	0,24
Mortalidad por cáncer de cuello uterino	–0,81	0,24	1,00

En conjunto, los resultados muestran que, aunque Chile presenta los mejores indicadores de esperanza de vida y menores tasas de cáncer, y Colombia y Nicaragua exhiben contextos más heterogéneos, el nivel del gasto público no explica por sí solo las diferencias observadas. La fuerte asociación inversa entre esperanza de vida e indicadores de cáncer sugiere un impacto significativo de esta enfermedad en la longevidad femenina. Las débiles correlaciones con el gasto evidencian la necesidad de evaluar la eficiencia, la focalización y el respaldo normativo de las políticas preventivas.

## Discusión

Los hallazgos del análisis estadístico ponen de manifiesto una situación preocupante para algunos países de América Latina, particularmente en relación con la incidencia y la mortalidad por cáncer de cuello uterino. Nicaragua, por ejemplo, mantiene una de las tasas más altas de incidencia y mortalidad en la región, con 20,6 y 11,5 por 100.000 mujeres, respectivamente, lo cual contrasta fuertemente con los niveles observados en Chile y en Colombia. Esta diferencia es consistente con estudios previos que han evidenciado las debilidades estructurales del sistema de salud nicaragüense, en particular en cuanto a cobertura, acceso y calidad de servicios de prevención y cribado^5^, ^20^.

En contraste, Chile muestra los mejores resultados en longevidad y control del cáncer cervicouterino, lo cual está respaldado por un marco normativo sólido como el plan AUGE (Acceso Universal con Garantías Explícitas), que garantiza la cobertura del cribado y el tratamiento oportuno del cáncer cervical como una prioridad de salud pública (Ministerio de Salud de Chile, 2020). Este tipo de legislación obliga al Estado a garantizar la detección precoz, asegurando pruebas de Papanicolaou, seguimiento y tratamiento sin barreras económicas, lo que ha contribuido a la disminución sostenida de la mortalidad en ese país.

Colombia ocupa un lugar intermedio, con avances significativos en el marco legal y en la implementación de programas como el del Fondo Colombiano de Enfermedades de Alto Costo, que monitoriza e implementa estrategias de control de cáncer en mujeres. Sin embargo, el país aún enfrenta brechas importantes en cobertura geográfica, equidad en el acceso a tecnologías de diagnóstico y adherencia a protocolos de seguimiento (Fondo Colombiano de Enfermedades de Alto Costo, 2024). La literatura también resalta que las diferencias regionales en Colombia, así como las barreras socioculturales y económicas, afectan la efectividad de los programas de prevención^2^, ^11^, ^14^.

Por otra parte, la atención primaria es el primer nivel de contacto y juega un rol clave en la identificación precoz de lesiones cervicales. Se recomienda la estandarización de protocolos de cribado en centros comunitarios, incluyendo el uso de pruebas autoadministradas de VPH para mejorar la accesibilidad en zonas rurales y marginadas^18^. Así mismo, el fortalecimiento de la capacitación de personal de salud y el registro sistemático de resultados facilitarán el seguimiento oportuno de casos.

Al analizar el impacto del gasto público en salud, la correlación débil entre este indicador y la mortalidad o incidencia del cáncer sugiere que no solo importa cuánto se invierte, sino cómo se distribuyen los recursos. Países como Nicaragua han incrementado gradualmente su gasto, pero sin una transformación estructural del modelo de atención y sin legislación robusta que garantice servicios universales y sostenibles^5^. Por su parte, la comparación entre Chile y Colombia muestra cómo un sistema con mayor regulación pública (como el chileno) puede generar mejores resultados en salud frente a uno más fragmentado y mixto, como el colombiano^6^, ^12^.

Desde una perspectiva legislativa internacional, es crucial recordar que la OMS y la OPS han lanzado iniciativas globales para la eliminación del cáncer de cuello uterino como problema de salud pública. Estas estrategias instan a los países a cumplir metas de vacunación, cribado y tratamiento con cobertura superior al 90% para alcanzar dicha eliminación^3^, ^4^, ^20^, ^21^. La falta de cumplimiento de estos estándares en países con altos niveles de desigualdad como Nicaragua representa una barrera estructural que debe ser abordada con reformas legislativas profundas y con el acompañamiento técnico y financiero de organismos internacionales.

Dentro de las limitaciones del estudio vale la pena resaltar que la naturaleza ecológica del diseño impide atribuir relaciones causales a nivel individual y puede enmascarar heterogeneidad subnacional. Además, el manejo de datos faltantes mediante *complete-case analysis* podría sesgar resultados si la ausencia de información no fue aleatoria. Tampoco se incluyeron variables como cobertura real de vacunación contra VPH o tasas de cribado por grupos etarios, lo que limitaría la comprensión completa de los determinantes de los indicadores. Por lo tanto, sería valioso realizar estudios cualitativos que exploren las percepciones de usuarias y profesionales sobre barreras y facilitadores del cribado. Además, evaluar la coste-efectividad de diferentes estrategias de cribado (citología vs. VPH) en contextos de recursos limitados proporcionaría evidencia para optimizar la asignación de presupuesto. Finalmente, estudios subnacionales permitirían identificar focos de alta vulnerabilidad y diseñar intervenciones coste-efectivas.

## Recomendaciones

Con base en estos hallazgos, se plantean tres recomendaciones clave:

• Fortalecer la legislación nacional en salud para garantizar el acceso universal y gratuito a servicios de prevención del cáncer de cuello uterino, particularmente cribado con pruebas sensibles como el VPH y seguimiento oportuno de casos positivos. Esta recomendación es especialmente urgente para Nicaragua.
• Rediseñar el uso del gasto público en salud orientándolo a la atención primaria y a intervenciones coste-efectivas como vacunación y cribado, asegurando que el aumento del financiamiento se traduzca en resultados sanitarios tangibles, como ocurre en el modelo chileno.
• Fortalecer la cooperación internacional para lograr la meta regional de eliminación del cáncer cervical, movilizando recursos técnicos, humanos y normativos para cerrar las brechas entre países. La OPS y OMS deben continuar liderando estrategias conjuntas con enfoque diferencial, especialmente en territorios con alta vulnerabilidad.

## Conclusiones

La solidez del marco legal y la dirección estratégica del gasto público son fundamentales para la efectividad de los programas de prevención. La experiencia de Chile demuestra que, con un régimen normativo como el AUGE que garantiza cobertura de cribado y tratamiento sin barreras financieras, se logran reducciones sostenidas en incidencia y la mortalidad por cáncer cervical.

La carga del cáncer de cuello uterino afecta de manera directa la longevidad de la población femenina. La correlación muy fuerte y negativa entre esperanza de vida e incidencia (r = −0,97) y mortalidad (r = −0,81) indica que las mujeres en contextos de alta carga de enfermedad pierden años de vida saludable que podrían prevenirse.

Un enfoque integral y con énfasis en equidad y atención primaria es clave para alcanzar las metas 90-70-90 de la OMS/OPS para cerrar brechas en países con sistemas fragmentados o con barreras socioculturales; es necesario combinar pruebas autoadministradas de VPH, estandarización de protocolos en atención primaria, monitorización rigurosa de indicadores de cobertura y estrategias interculturales que faciliten el acceso en poblaciones vulnerables.

**Puntos clave**

**Lo conocido sobre el tema**

• El cáncer cervicouterino sigue siendo una de las principales causas de muerte prevenible entre mujeres en América Latina.
• Se sabía que las diferencias en gasto público y cobertura sanitaria influyen en la detección temprana y en la mortalidad.

**Qué aporta este estudio**

• Este estudio compara por primera vez gasto, marco legal y resultados en Chile, Colombia y Nicaragua durante 2012-2022.
• Se encontró que la fuerza del marco normativo y la eficiencia del gasto explican más los resultados que el nivel de inversión.
• Se recomienda fortalecer la legislación, priorizar la atención primaria y ampliar el cribado y la vacunación para eliminar la enfermedad.

## Financiación

Los autores declaran que para la presente investigación ha contado con el aval y el apoyo de la Universidad El Bosque.

## Consideraciones éticas

Al basarse en datos secundarios agregados y de acceso público, este estudio no requirió aprobación de comité de ética ni consentimiento informado. No se manejaron datos individuales ni identificables.

## Conflicto de intereses

Los autores declaran no tener ningún conflicto de intereses.

## Agradecimientos

Al grupo de investigación de Cuidado de la salud y calidad de Vida adscrito a la facultad de Enfermería de la Universidad El Bosque.
